# Fitness Apps, Live Streaming Workout Classes, and Virtual Reality Fitness for Physical Activity During the COVID-19 Lockdown: An Empirical Study

**DOI:** 10.3389/fpubh.2022.852311

**Published:** 2022-06-22

**Authors:** Ru Liu, Rashid Menhas, Jianhui Dai, Zulkaif Ahmed Saqib, Xiang Peng

**Affiliations:** ^1^College of Physical Education, Hunan City University, Yiyang, China; ^2^Research Center of Sports Social Sciences, College of Physical Education and Sports, Soochow University, Suzhou, China; ^3^College of Physical Education and Sports, Soochow University, Suzhou, China; ^4^College of Management, Shenzhen University, Shenzhen, China

**Keywords:** COVID-19, lockdown, smart applications, mediating role, physical activity

## Abstract

**Background:**

Physical activity is an essential need of the human body that helps improve the physical fitness of an individual and creates a positive impact on overall wellbeing. Smartphone applications play an essential role in providing several benefits to consumers by offering various capabilities in terms of health and fitness.COVID-19 preventive measures shut down public places, and people cannot go to the gym and parks for physical activity. Smart applications for physical activity are an effective way to keep active while staying at home.

**Objective:**

The objective of the present study was to assess the mediating role of the e-platforms physical activity among the Chinese people in China during the COVID-19 lockdown.

**Method:**

The participants in this study were Chinese citizens living in home isolation during the early stages of the epidemic in China. The primary data was collected via an online survey using a convenience sample strategy in accordance with the study purpose. The collected data were cleaned by using the SPSS-25 statistical software. SmartPLS 3.0 software was used to investigate the suggested study framework utilizing the structural equation modeling technique.

**Results:**

Descriptive statistics shows that the ratio of gender includes 49% (*n* = 2,626) male and 51% females in the entire sample. SEM results show that all hypotheses (H1: β = 0.497, *T* = 43.068, *P* = <0.001; H2: β = 0.498, *T* = 41.078, *P* = <0.001; H3: β = 0.498, *T* = 41.078, *P* = <0.001; H4: β = 0.471, *T* = 39.103, *P* = <0.001; H5: β = 0.468, *T* = 42.633, *P* = <0.001; H6: β = 0.251, *T* = 11.212, *P* = <0.001; H7: β = 0.367, *T* = 16.032, *P* = <0.001; H8: β = 0.170, *T* = 13.750, *P* = <0.001; H9: β = 0.125, *T* = 10.604, *P* = <0.001; H10: β = 0.173, *T* = 14.842, *P* = <0.001) were statistically confirmed.

**Conclusion:**

In COVID-19, when there are limited physical activity resources, smart applications play an essential role as an alternative to gyms and change people's perspective regarding the adoption of health and fitness. Smart applications have made exercise and physical activity accessible and convenient to adopt.

## Introduction

The outbreak of COVID-19 emerged in China in December 2019 and spread worldwide. It has affected more than 179 M people and resulted in 3.08 M deaths worldwide. Due to COVID-19, there were lockdown conditions due to which people had to stay at home. In addition, everyone was under quarantine which was applied to alleviate the additional risk of contracting this virus among individuals. Physical activity is an essential need of the human body that helps improve the physical fitness of an individual and creates a positive impact on their mental health. It helps individuals combat various diseases and reduces stress levels (1). The imposition of lockdown and self-isolation across the globe has affected people's health badly and created poor people's health conditions, including obesity, anxiety and depression, impaired cognition, psychological or stress-related disorders, unusual eating and sleeping patterns, and a decrease in motor ability. These poor conditions have led to people's poor quality of life in the state of quarantine. However, among all the effects, the most critical impact of the spread of this pandemic has been psychological distress. The pandemic has increased the rate of psychological pain and psychiatric illness among people worldwide. This impact has disturbed the people's mental conditions, affected their physical condition of the people, and resulted in a poor lifestyle of the people in the time of the pandemic. Furthermore, this psychological distress has to be considered pandemic-related. It has created a wave of fear among people to contact this novel virus and lose their loved ones, creating future uncertainty (2).

Smartphone applications have started playing a significant role in society. They offer various home-based activities and provide a facility to stay fit and healthy while staying at home. The downloading rate of smart health applications is so high, especially in the times of COVID-19. But the rate of uninstallation is also increased as many people decide to discontinue these applications for several reasons, such as the burden of data entry, hidden costs, and lack of motivation interest. Although these smart applications promote a healthy lifestyle, there has been a gap between the content related to exercise and weight loss and guidelines based on evidence. Some people do not find this physical activity effective, but some think it is beneficial. On the other hand, researchers believe that innovative applications for physical activity are practical and helpful in short-term use and not reliable for long-term use (3–6). Smart applications related to health and fitness are divided into disease management applications and behavioral change in health. Disease management applications are smart systems that help manage the patients' medication and prevent any complications. These apps are designed to look at the patient's data and promote self-care. On the other hand, behavioral change in health is the application that helps to promote physical activity and fitness for all ages (7). Virtual reality (VR) applications have played an essential role in health and wellness in this era of technology. There are adverse side effects of using technology (social media, smartphones, etc.) on health and fitness. However, there are some positive effects if the technology is used properly. There is virtual reality (VR) applications that help to promote exercise or physical activity. Physical activity through these applications is far more different than working out in gyms (8, 9).

However, smart applications have also created some problems and challenges for users, such as people dealing with being overweight. The people using these apps for weight loss find it tough to change their routine according to the suggestions. They are suggested to use low-calorie food, give up many food items, and adopt different advanced exercises, but they are not used to this routine. Hence, they find it challenging to adopt as they integrate the new habit into their daily routine. Another issue is the knowledge gap since these apps do not have a complete understanding of the user's situation and do not know whether the practice recommended by the apps is appropriate and beneficial for them (10). Physical inactivity is very harmful to an individual's health. It gives birth to various diseases and physical and mental issues such as depression, anxiety, stress, obesity, poor concentration, and many others. Physical inactivity makes a person physically, mentally, and emotionally weak. Physical activity and fitness are solutions to combat or prevent many diseases and are required for a healthy lifestyle. The present time is the era of smart technology, which has conquered almost every field, making the work easier to complete. The same has happened in fitness, where smartphone applications have played an essential role in providing several benefits to consumers by offering various health and fitness capabilities. These applications facilitate the consumers across multiple fitness exercises such as physical exercise, nutritional food diet, weight loss, healthy food choices, and workout routine, and help prevent many diseases like cardiovascular diseases, diabetes, and heart failure. The market of smartphone fitness applications is rapidly growing and assisting consumers in giving attention to their health conveniently (1). Physical activity is significant for a better and healthy life. In COVID-19, when there are limited physical activity resources, smart applications play an essential role as an alternative to gyms and change people's perspectives regarding the adoption of health and fitness. These smart applications have made exercise and physical activity accessible and convenient to adopt. Therefore, these smart applications have been beneficial in promoting and improving the health and fitness of people. The objective of the present study was to assess the mediating role of fitness apps, live streaming workout classes, and virtual reality fitness for physical activity during the COVID-19 lockdown among the Chinese people in China. The study hypotheses are given below.

### Proposed Hypothesis

H1: The usage of fitness applications is influenced favorably by the COVID-19 preventative measures (CPM).

H2: The live streaming workout classes (LSWc) are positively influenced by the COVID19 preventive measures (CPM).

H3: The use of virtual reality fitness (VRF) for physical activity is positively influenced by the COVID19 preventive measures (CPM).

H4: COVID19 preventive measures (CPM) positively influence physical activities (PA's).

H5: Fitness apps (FA's) positively affect physical activities (PA's).

H6: Physical activities (PA's) positively impacted by live-streaming workout classes (LSWc).

H7: Physical activities (PA's) are positively impacted by virtual reality fitness (VRF).

H8: The relationship between COVID19 preventive measures (CPM) and physical activities (PA's) is positively mediated by fitness apps (FA's).

H9: The relationship between COVID19 preventive measures (CPM) and physical activities (PA's) is positively mediated by live-streaming workout classes (LSWc).

H10: The relationship between COVID19 preventive measures (CPM) and physical activities (PA's) is positively mediated by virtual reality fitness (VRF).

## Materials and Methods

The method section comprises four parts; study locale, participants, instrument and data collection, measures, and analysis.

### Study Locale

The current study was conducted in China. The study upheld the standards of the World Medical Helsinki Policy. Before the final data collection, the study was approved by the Ethical Committee of Soochow University, Suzhou, Jiangsu. The study participants gave their informed consent prior to the start of the final data collection.

### Participants and Data Collection

The participants in this study were Chinese citizens living in home isolation during the early stages of the epidemic in China. In order to obtain primary data for the study, a convenience sampling strategy was adopted. Convenience sampling is the most common type of non-probability sampling, and it focuses on getting responses from persons who are 'convenient' for the investigator to contact. The current study used convenience sampling due to time limitations, cheap sampling method, easy to use, and getting results quickly. A pre-testing of 75 respondents was conducted before the final survey to determine the reliability. Between January and March of 2020, an online survey was done. A total of 5,600 questionnaires were sent to the targeted population. About 5,450 (97.32%) questionnaires were responded to by the eligible individuals for participation in the study. After a quality check of the data, a total of 5,351 respondents' response was analyzed (see Table 1).

**Table 1:** Data quality check distribution.

**Items**	**Frequency/percentage**
The questionnaires sent to the study participants	5,600 (100.0%)
Questionnaire responded by the study participants	5,450 (97.32%)
Questionnaire not responded	150 (2.68%)
Incomplete excluded questionnaire	99 (1.77%)
The questionnaire included in the final data analysis	5,351 (95.55%)

### Questionnaire

A well-designed questionnaire was used to obtain primary data. Demographic questions were related to gender, age, education, occupation, and marital status. Closed-ended, open-ended, and Likert scale items were used in the questionnaire (see Supplementary Table 1).

### Study Variables

#### E-Platforms for Physical Activity and Health Wellbeing

Regular exercise is a natural remedy for a variety of ailments and strengthens the immune system of the human body. The World Health Organization issued specific instructions for remaining active at home throughout the lockdown time (11). Physical activity-related questions included participation in physical activity, frequency of physical activity during the COVID-19 lockdown, location of physical activity during the COVID-19 lockdown, indoor, and duration of physical activity during the COVID-19 lockdown (See Supplementary Table 1). Effective interventions to increase daily fitness and physical health are needed. The usage of cell phones while exercising is one potential trend (12). App use in leisure jogging is robust and expanding, and a variety of apps have been designed to help people with their sporting activities (13). Physical exercise or physical activity has a very significant and essential role in the life of a person. It helps improve an individual's mental and physical health and other benefits in the long term. It also helps reduce many disease risks like those who include physical exercise in their routine face fewer risks of cardiovascular, cancer, and type 2 diabetes diseases. The fitness and health apps have created drastic changes in health and wellbeing. Public spaces such as gyms, squares, parks, and walking trails were closed during the COVID-19 lockdown. Physical activity is a natural way to keep the immune system strong against various diseases (14). The e-platforms related questions were asked from the study participants to assess the mediating role of physical activity and health wellbeing (See Supplementary Table 1). According to published findings, smartphone physical exercise treatments have a small-to-moderate positive effect on physical activity indices. The use of applications, which are mainly focused on behavior transfer approaches, would enhance brain processes that would encourage fitness and regular exercise (15).

#### COVID-19 Prevention Measures

COVID-19 is a newly discovered infectious disease affecting society globally in every aspect of life. Health experts, especially the WHO, announced various measures to prevent the COVID-19 transmission for health and wellbeing. Preventive measures related to the COVID-19 include washing hands, keeping social distance, wearing masks, staying at home, darning lockdown, quarantine, doing physical activity while staying at home, and monitoring health (11). COVID-19 prevention-related questions were based upon the Likert scale and asked the respondents how closely they agreed and followed the measures during the lockdown period (See Supplementary Table 1). Exercise has a substantial impact on the immune system. COVID-19 has unfavorable impacts on those who do not engage in regular physical exercise (16). Increased body exercise is thought to lessen a human's vulnerability to COVID-19 infection.

### Data Analysis

To display the demographic data and clean and prepare the data for testing all hypotheses, SPSS-25 statistical software was utilized. For this work, the proposed research framework was explored utilizing the structural equation modeling (SEM) technique, which was implemented using SmartPLS 3.0 software. In terms of constructs and indicators, the partial least squares (PLS) SEM approach was employed (17). The SEM approach is used to analyze the model using discriminant and convergent validity and factor loadings to generate average variance extracted values for each construct. As a result, it's a multivariate analytic approach for analyzing the conceptual model's various connections between variables.

## Results

### Study Participants Characteristics

According to Table 2 statistics, the ratio of gender includes 49% (*n* = 2,626) male and 51% females of the full sample. For age, 2,848 (53.22% of 5,351) respondents belonged to the 20–29 age group; 30–39 had 25.70% (*n* = 1,376) respondents. The remaining 20% of respondents belong to three groups (the 40–49 age group have 527 respondents) (50–59 age group have 444 respondents), and (60+ age group have 156 respondents). The education profile of respondents was included with college graduation 31.34% (*n* = 1,677), university graduate 42.46% (*n* = 2,272), and others 26.20% (*n* = 1,402). Further, most of total respondents were earning privately with 42.7% of 5,351 (*n* = 2,287), 19.8% of 5,351 were students, 16.8% of 5,351 (*n* = 897) were self-employed, government employees were 11.7 % of 5,351 (*n* = 628), the retired respondents were less with 0.7% (*n* = 39), 1% (*n* = 48) have no occupation, and 7.3% of 5,351 (*n* = 390) have nameless occupations. More, 62.5% of 5,351 (*n* = 4,347) respondents were married, 35% of 5,351 (*n* = 1,871) were single/unmarried, and 133 respondents were belonging to nameless marital option.

**Table 2:** Demographic distribution of the study population (*N*-5,351).

**Variables**	**Categorization**	**Frequency**	**Percent**
Gender	Male	2,626	49.1%
	Female	2,725	50.9%
Age	20–29	2,848	53.22%
	30–39	1,376	25.70%
	40–49	527	9.8%
	50–59	444	8.3%
	60+	156	2.9%
Education	College graduate	1,677	31.34%
	University graduate	2,272	42.46%
	Others	1,402	26.20%
Occupation	Government employee	628	11.7%
	Private companies employee	2,287	42.7%
	Self-employed	897	16.8%
	Retired	39	0.7%
	Student	1,062	19.8%
	No occupation	48	1.0%
	Others	390	7.3%
Marital status	Married	3,347	62.5%
	Unmarried (single)	1871	35%
	Others	133	2.5%

### Measurement of the Research Framework

To examine the construct reliability and validity, the factor loadings, average variance extracted (AVE), scale composite reliability (SCR), and convergent and discriminant validity are used. The factor loadings of all items were found between 0.651 and 0.942, and loading values were significant at a 5% level (see Table 3). Several literary works regarding the acceptance level of loading values for better results, and researchers suggested loading values should be equal to or higher than 0.50 (18, 19). Results indicate that loading values were found in the criteria and acceptable. A reasonable standard for SCR value is equal to 0.60 and up (20). Results shows that SCR values are 0.834, 0.953, 0.945, 0.946, and 0.950 (see Table 3) which are acceptable. To examine the reliability, the threshold limit of Cronbach's alpha is higher than 0.70, and the calculated Cronbach's alpha values for all constructs are 0.755, 0.935, 0.923, 0.924, and 0.930. More, variance inflation factor (VIF) values were calculated and used to check the multicollinearity among variables. Multicollinearity affects the path coefficients which are reviewed from VIF values. The standard for VIF values is <5.0 to check the multicollinearity (21). The VIF values are <5.0 (see Table 3), which means there is no multicollinearity issue with the data.

**Table 3:** Results for measurement of the conceptual framework (*N*-5,351).

**Constructs**	**Items**	**Factor loadings**	**Std. D**	***T*-values**	**VIF**	**SCR values**	**AVE values**	**Cronbach's Alpha**
Covid-19 preventive measures	CPM1	0.693	0.011	61.91	1.35	0.834	0.502	0.755
	CPM2	0.741	0.010	76.82	1.44			
	CPM3	0.702	0.015	48.03	1.48			
	CPM4	0.751	0.008	96.74	1.34			
	CPM5	0.651	0.014	46.39	1.31			
Fitness app	FA1	0.942	0.002	428.01	4.79	0.953	0.837	0.935
	FA2	0.908	0.003	288.14	3.40			
	FA3	0.900	0.003	258.00	3.15			
	FA4	0.909	0.003	286.20	3.42			
Live-streaming workout classes	LSWc1	0.900	0.003	280.56	3.04	0.945	0.812	0.923
	LSWc2	0.901	0.003	269.34	3.06			
	LSWc3	0.899	0.003	283.87	3.02			
	LSWc4	0.905	0.003	300.14	3.18			
Physical activity	PA1	0.897	0.003	260.08	2.97	0.946	0.814	0.924
	PA2	0.904	0.003	302.55	3.16			
	PA3	0.903	0.003	299.11	3.14			
	PA4	0.905	0.003	302.62	3.18			
Virtual reality fitness	VRF1	0.906	0.003	285.14	3.24	0.950	0.826	0.930
	VRF2	0.912	0.003	311.83	3.39			
	VRF3	0.910	0.003	306.61	3.33			
	VRF4	0.907	0.003	294.12	3.27			

Table 4 demonstrates the results for discriminant validity regarding variables. AVE is used to examine the discriminant and convergent validity by following the Fornell-Larcker Criteria. The threshold for the AVE value is above 0.50 (20). The calculated AVEs are higher than 0.50, which were found to be adequate. Therefore, discriminant validity could be achieved by comparing AVEs values with correlation coefficients where the square root for each AVE should be higher than the correlation refection. In Table 4, the off-diagonal values are correlation coefficients that are less than diagonal values (AVEs), which shows that the discriminant validity is achieved. More, convergent validity is based on factor loading values and AVE values. The AVEs and factor loadings are within limits, meaning latent variables have more than 50% variance regarding observed variables.

**Table 4:** Constructs discriminant validity by following Fornell-Larcker Criteria.

**Constructs**	**CPM**	**FA**	**LSWc**	**PA**	**VRF**
Covid-19 preventive measures	**0.709**				
Fitness apps	0.497	**0.915**			
Live streaming workout classes	0.498	0.888	**0.901**		
Physical activity	0.498	0.884	0.879	**0.902**	
Virtual reality fitness	0.471	0.872	0.886	0.887	**0.909**

*Bold values denote correlations (off-diagonal elements) and square root of the AVEs (diagonal elements)*.

### Model Fitness

The measurement of model fit is designed, which is based on the Standardized Root Mean Square Residual (SRMR) value, Chi-Square (χ^2^), normed fit index (NFI), unweighted least squares discrepancy (d_ULS), and geodesic discrepancy (d_G) values (18). The standard value of SRMR is <0.08, indicating a good fit and suitability of the structural model. The final results for model fitness indicate that the SRMR value is 0.043 and acceptable. The threshold for the NFI value is higher than 0.80, and the found value for NFI is 0.947 by this work. According to Table 5, the value of χ^2^ is 5459.225, and the results indicate that the model's fitness is good and acceptable. Two distinct methods of computing the discrepancy are d_ULS and d _G. The significance levels for these discrepancy values are provided by the bootstrap method. The d_G criterion is based on eigenvalue calculations in PLS-SEM. The exact model fit measures allow results to be interpreted by bootstrap findings (22).

**Table 5:** Model fit summary.

**Statistical tests**	**Estimated model**
SRMR	0.043
d_ULS	0.418
d_G	0.184
Chi-Square (χ2),	5,479.225
NFI	0.947

*SRMR, standardized-root-mean-square-residual; d_ULS, unweighted least squares discrepancy; d_G, geodesic discrepancy; NFI, normed fit index*.

### Structural Equation Modeling

The structural model is examined by achieving the validity of the measurement model, and the structural model is confirmed (Reference). The SEM approach tests the proposed hypothesis in the conceptual framework. SEM approach accepts or rejects the proposed hypothesis based on the significance level (5% level) of relationships13. Six direct and four indirect assumptions are presented in the structural model, and the structural model was examined by employing the bootstrapping method (See Table 6). The results for the proposed hypotheses are presented in Table 5 with path coefficients (β), *P*-values, and *T*-values. Surprisingly, SEM results show that all assumptions were statistically significant at *P* = <0.001. In other words, overall Covid-19 preventive measurement (CPM) has a substantial effect on human physical activities PA. According to Hypothesis H1, H2, and H3, the impact of CPM on the use of advanced technologies such as fitness apps (FA), live streaming workout classes (LSWc), and virtual reality fitness (VRF) is proposed as positive. H1 indicates that CPM positively influences FA with (β = 0.497, *T* = 43.068, and *P* = <0.001) values, which confirmed the acceptance. The estimated values (β = 0.498, *T* = 41.078, and *P* = <0.001) of H2 indicate that the effect of CPM on LSWc is positive and statistically confirmed. According to H3, VRF is positively affected by CPM (β = 0.498, *T* = 41.078, and *P* = <0.001), and H3 is accepted. Further, the direct effect of CPM on PA as H4 is also included. The results for H4 indicate that CPM has a positive impact on PA with (β = 0.471, *T* = 39.103, and *P* = <0.001), which means the proposed H4 is supported. The effect of using technologies such as FA, LSWc, and VRF toward PA is presented as H5, H6, and H7, respectively. Final results indicate that H5, H6, and H7 are supportive, and the effect of FA, LSWc, and VRF on PA are positive with (β = 0.468, *T* = 42.633, and *P* = <0.001), (β = 0.251, *T* = 11.212, and *P* = <0.001), and (β = 0.367, *T* = 16.032, and *P* = <0.001), respectively. To examine the indirect relationships among CPM and PA, hypotheses H8, H9, and H10 are proposed as the positive mediating role of FA, LSWc, and VRF. SEM results show that the indirect effect of FA, LSWc, and VRF is positive and supportive with (β = 0.170, *T* = 13.750, and *P* = <0.001) for H8, (β = 0.125, *T* = 10.604, and *P* = <0.001) for H9, and (β = 0.173, *T* = 14.842, and *P* = <0.001) for H10.

**Table 6:** Structural results for proposed hypotheses.

**Direct and indirect hypotheses**	**Beta**	***T*-value**	***P*-values**	**Results**
H1 = CPM -> FA	0.497	43.068	0.001	Confirmed
H2 = CPM -> LSWc	0.498	41.078	0.001	Confirmed
H3 = CPM -> VRF	0.471	39.103	0.001	Confirmed
H4 = CPM -> PA	0.468	42.633	0.001	Confirmed
H5 = FA -> PA	0.341	14.357	0.001	Confirmed
H6 = LSWc -> PA	0.251	11.212	0.001	Confirmed
H7 = VRF -> PA	0.367	16.032	0.001	Confirmed
H8 = CPM -> FA -> PA	0.170	13.750	0.001	Confirmed
H9 = CPM -> LSWc -> PA	0.125	10.604	0.001	Confirmed
H10 = CPM -> VRF -> PA	0.173	14.842	0.001	Confirmed

*CPM, COVID-19 Preventive Measures; FA, Fitness Apps; LWSc, Live streaming workout classes; VRF, Virtual reality fitness*.

## Discussion

Physical exercise plays a vital role in the life of a person. It helps to improve an individual's mental and physical health and other benefits in the long term. It also helps reduce many disease risks like those who include physical exercise in their routine face fewer risks of cardiovascular, cancer, and type 2 diabetes diseases. Those who do physical activity regularly can improve their lives and reduce the risk of chronic illness. It helps enhance sleep quality, manage weight, improve memory and brain function in people of all ages, improves joint stiffness, and maintain balance strength muscles (23). The fourth-largest factor which is contributing to mortality is physical inactivity (24). In 2017, activated mobile phones outnumbered residents globally, with over (63%) of the adult population possessing at least one smartphone (25, 26). Smart applications play a vital role in promoting physical activity trends among the inactive population because it's a promising way to enhance physical activity. The COVID-19 pandemic has negatively impacted physical activity across the globe because of the closure of public facilities for physical exercise. Physical activity is one of the best natural measures against various diseases to keep the immune system strong (12, 27). Smart applications, including numerous digital platforms, are the best ways to maintain physical activity while staying at home during preventive measures the COVID-19, such as lockdown and social distancing. According to the statistics of our study, more females than men use smart applications for physical activity and health wellbeing, and these results are in line with previous research findings (28, 29). People's attention has been drawn to the need for physical exercise as a result of the COVID-19 outbreak. Throughout the outbreak, the mainstream media has stressed the advantages of regular physical exercise on overall fitness and persons with mild coronavirus infection. Physical exercise boosts immunological surveillance, acts as an anti-inflammatory agent, reduces the risk of developing a number of chronic diseases, and promotes overall fitness and sickness protection and response (30).

### Fitness Apps and Physical Activity

Smartphones have reached unprecedented levels of worldwide appeal. In China (73.1%), of youngsters have a smartphone (31). Smart applications and digital platforms have innovative features such as a built-in accelerometer, global positioning, and internet connectivity, providing a constructive physical activity intervention for health and wellbeing (32). The results of our study (β = 0.497, *T* = 43.068, and *P* = <0.001) confirm that smart applications such as fitness apps are positively influenced by preventive measures against the COVID-19. People highly acknowledge the importance of physical activity for health and wellbeing during the COVID-19 preventive measures and adopt various smart and digital platforms for active living through physical activity. Similar results were reported by Lynch et al. (33), and they found that the FT interventions positively impact physical activities like step count compared with the control condition. From WHO and the US Center for Disease Control and Prevention, the telehealth information was collected, and PA's data for the period of lockdown was also reviewed. And all those mHealth applications are enlisted that are used in the physical activity promotion at home. After observing these things, they concluded that mHealth applications could physically enhance health and be utilized during lockdown at home (34). Health and fitness apps mitigate the negative impact of the COVID-19 preventive measures on physical activity. Our study participants used smart applications to maintain their regular physical activity level and reduce the physical inactivity which was caused due to travel restrictions. In line with our results, a US study reported the same results conducted to assess the changes in physical activity during the COVID-19. It was concluded that the use of physical activity apps might reduce the impact of the decline in physical activity and can be helpful in reducing the health risks (35). A healthy lifestyle requires regular bodily exercise through advanced approaches, which further create motivations for active living. Smartphone apps could be an excellent tool for promoting physical activity and overall wellness (36).

### Physical Activity by Adopting Live Streaming Workout Classes

Traditional PA settings and facilities cannot use during the home isolation. The usage of digital platforms during the unexpected pandemic crisis may shed light on their prospective role in assisting people in meeting their MVPA, MSE, and combined physical activity needs (37). Our findings show that CPM toward LSWc is positive and statistically confirmed (β = 0.498, *T* = 41.078, and *P* = <0.001). The LSWc has a positive role in promoting physical activity and achieving the recommended level of physical activity during the CPM. Parker et al. (38) observed similar outcomes and endorsed live-streaming fitness sessions for physical activity as part of the preventative measures for the COVID-19. Live streaming on digital platforms such as YouTube during the COVID-19 lockdown has promoted physical activity and muscle-strengthening exercises among the home confined population. Adolescents can benefit from digital tools that encourage and support physical exercise (39). Fitness influencers and online exercise clubs allow their viewers and subscribers to participate in workouts through live streaming. Online platforms promote health and fitness by showcasing lifestyle sports and fitness trends and disseminating health and fitness messaging (40). The COVID-19 epidemic was a significant driver for home-based digital sports activities. All sports and fitness activities that are primarily assisted by or involve digital media (smartphone apps, DVDs, digital technologies, on-demand videos, and live streaming) are referred to as digital sports activities that gained popularity during the COVID-19 restrictions for active participation living (41). Digital sports through live streaming also play a vital role in behavioral change regarding physical activity and wellness. In public health, digital sports through live streaming encourage people to stay active during the pandemic restrictions (7, 42, 43). Srivastav et al. (34) published a report to discuss mHealth applications and VR systems for enhancing PA at home via a motivating and interactive digital environment. From WHO and the US Center for Disease Control and Prevention, the telehealth information was collected, and PA's data for the period of lockdown was also reviewed. And all those mHealth applications are enlisted that are used in the physical activity promotion at home. It has been found that mHealth applications and VR systems enhance PA improving people's lives.

### Virtual Reality Fitness and Physical Activity

Virtual reality workout means less body pain and more calories burned, so virtual reality has a significant impact on exercise in today's world compared to the actual workout. Most people worldwide have health issues because of obesity and are not attracted to older ways of working out. Whereas our new generation grew up with the technology and is attracted to virtual reality, they spend most of their time on laptops and PCs. Most people will be interested in virtual reality rather than actual workouts. They relate their lives to virtual realities. If they choose between a virtual reality workout and an actual workout, they will probably go for a virtual reality workout because they won'*T* have to go out for that and enjoy the workout (44). The statistics of our study (β = 0.170, *T* = 13.750; *P* = <0.001; β = 0.125, *T* = 10.604 *P* = <0.001; β = 0.173, *T* = 14.842, *P* = <0.001) show that VRF has a positive impact on PA during the CPM implementations. Virtual reality fitness apps are based upon virtual environment exergames. Exercise with video games that rely on technology refers to an exergame that measures body movements during physical activity (45). Because VR sensors can detect body motions and immerse a user's feelings, VR immersion is a new trend in exergames (46). VRF has been acknowledged as a novel strategy to enhance physical activity and healthy habits. It is rapidly being employed in promoting health. Virtual reality (VR) applications play an important role in health and fitness in this era of technology. In the domains of biomechanics and population health, the development of virtual reality technology and its usefulness during workouts via its incorporation with common training apparatus and rehabilitative procedures have gotten a lot of attention (47, 48).

## Conclusion

In COVID-19, when there are limited physical activity resources, smart applications play an essential role as an alternative to gyms and change people's perspective regarding the adoption of health and fitness. Smart applications have made exercise and physical activity accessible and convenient to adopt. These smart applications have been beneficial in promoting and improving the health and fitness of people. Behavioral change in health is the unique feature of smart applications that helps to promote physical activity and fitness for all ages. The findings of our study show that smart applications have the potential to promote physical activity during a sudden pandemic. Smartphone applications have started playing a significant role in society as they offer various home-based activities and provide a facility to stay fit and healthy while staying at home.

## Limitations of the Study

The Chinese government has established a program called “Internet plus Exercise” to encourage people to live a more active lifestyle and improve their overall health. The participants in this study were Chinese citizens (+20 years old) who were in home isolation throughout the epidemic and engaged in physical exercise via internet platforms under the “Internet plus Exercise” intervention. The participant's participation in the study was voluntary. Under the non-probability sampling (NPS) strategy, the convenience sampling method was utilized to acquire data. The original data were collected by a self-administered survey, and the findings of convenience sampling cannot be applied to the entire population.

## Future Scope of the Study

A future study can be conducted jointly keeping in view the WHO guidelines about the use of the app for fitness and the Chinese government intervention “Internet plus Exercise” for physical activity in the context of the kind of exercise; mobility, strengthening, aerobic, anaerobic, the average time people used the products; per session, per week, overall.

## Data Availability Statement

The original contributions presented in the study are included in the article/Supplementary Material, further inquiries can be directed to the corresponding author/s.

## Ethics Statement

The studies involving human participants were reviewed and approved by the Ethical Committee of Soochow University, Suzhou, Jiangsu, approved the study before the final data collection. The patients/participants provided their written informed consent to participate in this study.

## Author Contributions

RM is the principal investigator, designed the study developed the hypothesis, conducted the survey, and wrote the final draft. ZS performed the statistical analysis. RM, RL, JD, and XP review and edit the final draft. All authors approved the final draft for publication.

## Funding

Hunan Province Educational Science Planning Project Lost and Redemption: Research on School Sports Justice in the Background of Sports Middle School Entrance Examination (XJK22QTW001).

## Conflict of Interest

The authors declare that the research was conducted in the absence of any commercial or financial relationships that could be construed as a potential conflict of interest.

## Publisher's Note

All claims expressed in this article are solely those of the authors and do not necessarily represent those of their affiliated organizations, or those of the publisher, the editors and the reviewers. Any product that may be evaluated in this article, or claim that may be made by its manufacturer, is not guaranteed or endorsed by the publisher.
