# Supplementary material for: Fitness Apps, Live Streaming Workout Classes, and Virtual Reality Fitness for Physical Activity During the COVID-19 Lockdown: An Empirical Study
Source: Front Public Health. 2022 Jun 22;10:852311. doi: 10.3389/fpubh.2022.852311 (PMC9257108; doi:10.3389/fpubh.2022.852311)
Supplement: Supplementary file 1 [file Table_1.DOCX]

**Questionnaire Questions**

| **Dimension** | **Variables** | **Description** | |
| --- | --- | --- | --- |
| Demographic Information | Gender | Male/Female | |
|  | Age | 20 – 29/ 30 – 39/ 40 – 49/ 50 – 59/ 60 + | |
|  | Education | College Graduate/ University Graduate/ Others | |
|  | Occupation | Government Employee/ Private Companies Employee/ Self-employed/ Retired/ Student/ No occupation/ Others | |
|  | Marital Status | Married/ Unmarried (single)/ Others | |
| **Dimension** | **Symbol** | **Items** | **Description** |
| COVID-19 Preventive Measures | CPM | CPM1 | Stay at home |
|  |  | CMP2 | Social distancing |
|  |  | CPM3 | Wearing facemask |
|  |  | CPM4 | Carry out self-declaration |
|  |  | CPM5 | Personal hygiene |
|  |  | CPM6 | Staying physically active at home |
|  |  | CPM7 | Avoid public gatherings |
|  |  | CPM8 | Quarantine |
| Fitness Apps | FA | FA1 | The use of fitness apps is helpful in the maintenance of physical wellness |
|  |  | FA2 | The use of a fitness app is helpful in the achievement of the required physical activity level |
|  |  | FA3 | The use of fitness apps is helpful in health monitoring |
|  |  | FA4 | The use of fitness apps is helpful in healthy and active living |
| Live Streaming Workout Classes | LSWC | LSWc1 | Live streaming workout classes help perform the physical activity at home |
|  |  | LSWc2 | Live streaming workout classes are helpful in the maintenance of physical wellness |
|  |  | LSWc3 | Live streaming workout classes are helpful in the achievement of the required physical activity level |
|  |  | LSWc4 | Live streaming workout classes are helpful in the healthy and active living |
| Virtual Reality Fitness | VRF | VRF1 | Virtual reality fitness helps participate in physical activity at home |
|  |  | VRF2 | Virtual reality fitness helps maintain physical wellness |
|  |  | VRF3 | Virtual reality fitness is helpful in the achievement of the required physical activity level |
|  |  | VRF4 | Virtual reality fitness is helpful in healthy and active living |
| Physical Activity | PA | PA1 | During the lockdown period, the use of fitness and health apps for physical activity is helpful |
|  |  | PA2 | During the lockdown period, you have an excellent motivation for physical activity because of using fitness apps, virtual reality fitness, and live streaming workout classes |
|  |  | PA3 | During the lockdown period, your physical activity level increases by using fitness and health apps |
|  |  | PA4 | Fitness apps, virtual reality fitness, and live streaming workout classes help you to achieve the fitness goal |
